# A Cross-Sectional Clinicomycological Study on Dermatophytosis: A Report From a Single Tertiary Healthcare Center in Eastern India

**DOI:** 10.7759/cureus.31728

**Published:** 2022-11-21

**Authors:** Dipmala Das, Himel Mondal, Asitava Deb Roy, Amit Anand, Prasanta Maiti, Atanu Ray

**Affiliations:** 1 Microbiology, All India Institute of Medical Sciences, Deoghar, IND; 2 Physiology, All India Institute of Medical Sciences, Deoghar, IND; 3 Pathology, All India Institute of Medical Sciences, Deoghar, IND; 4 Microbiology, Mata Gujri Memorial Medical College, Kishanganj, IND

**Keywords:** fungus, trichophyton mentagrophytes, tropical region, tinea corporis, trichophyton, tinea capitis, mycology, india, hygiene, dermatology

## Abstract

Background

Dermatophytosis is a public health concern in tropical countries. In India, a scalable number of dermatophytosis cases from multiple states are reported. In the eastern part of India, very few studies were published assessing the clinicomycological profiles of patients. Hence, we conducted this study to ascertain the clinicomycological profile of patients suffering from dermatophytosis with special reference to associated socio-environmental factors.

Materials and methods

This cross-sectional observational study was conducted in a tertiary care hospital situated in Bihar state of India from January 2021 to December 2021. We included a total of 330 patients of all age groups who were clinically diagnosed with superficial mycosis from the Department of Dermatology and sent for investigations to the Department of Microbiology. The collected specimens from the lesions were prepared with wet potassium hydroxide and examined under the microscope. Then, the specimens were inoculated and incubated at 25°C for up to four weeks. Fungal isolates were identified by gross appearance and microscopy if growth was observed.

Results

Among the 330 patients, 186 (56.4%) were males and 144 (43.6%) were females. The majority of the patients (54.5%) were from the low socioeconomic group and living in overcrowded places. Direct microscopy was positive in 198 (60%) patients, and culture was positive in 68 (20.61%) patients. The majority of the patients who were found positive in direct microscopy were from the age group of 21-30 years (39.9%), followed by 1-10 years (25.25%). A total of 92 (46.4%) cases were of tinea capitis, followed by 68 (34.3%) patients of tinea corporis. *Trichophyton *was the predominant fungus isolated, and *Trichophyton mentagrophytes *was the most common species (52.6%).

Conclusion

Tinea capitis was the most common provisionally diagnosed dermatophytosis in our tertiary care hospital in Bihar, an Indian state in its eastern zone. Low socioeconomic status and poor personal hygiene were the factors associated with the high prevalence of dermatophyte infections in this region of India. A detailed analysis of all these epidemiological factors is needed to limit the prevalence of dermatophytosis in tropical regions.

## Introduction

Dermatophytosis, commonly referred to as ringworm, is a public health concern around the globe with a high prevalence in tropical countries such as India. Although it is not a life-threatening disease, it is associated with numerous mental agonies and social stigma. Therefore, from a public health perspective, it deserves special attention [1].

Dermatophytosis is one of the common infections of keratinized nonliving tissues involving the epidermis, hair, and nails [2]. The three asexual genera, *Microsporum*, *Trichophyton*, and *Epidermophyton*, are the responsible fungus for the disease. Direct contact with infected humans or animals makes this fungus spread quickly [3]. Although the infection is noninvasive and does not warrant any emergency treatment in the majority of cases, itching is the major morbidity. The spread of the fungus is accelerated in a hot and humid climate and among people maintaining poor hygiene practices. Hence, the disease is still a public health issue in tropical countries such as India where healthcare access is limited [4].

There is an increasing trend of dermatophytosis in different parts of India [5-7]. However, studies from Eastern India are only a few, although there is a high prevalence of the disease in this area [7-10]. Hence, we designed this study to ascertain the clinicomycological profile of dermatophytosis with special emphasis on the associated socio-environmental factors in a semi-urban district of Eastern India.

## Materials and methods

Type and settings

**Figure 1:**
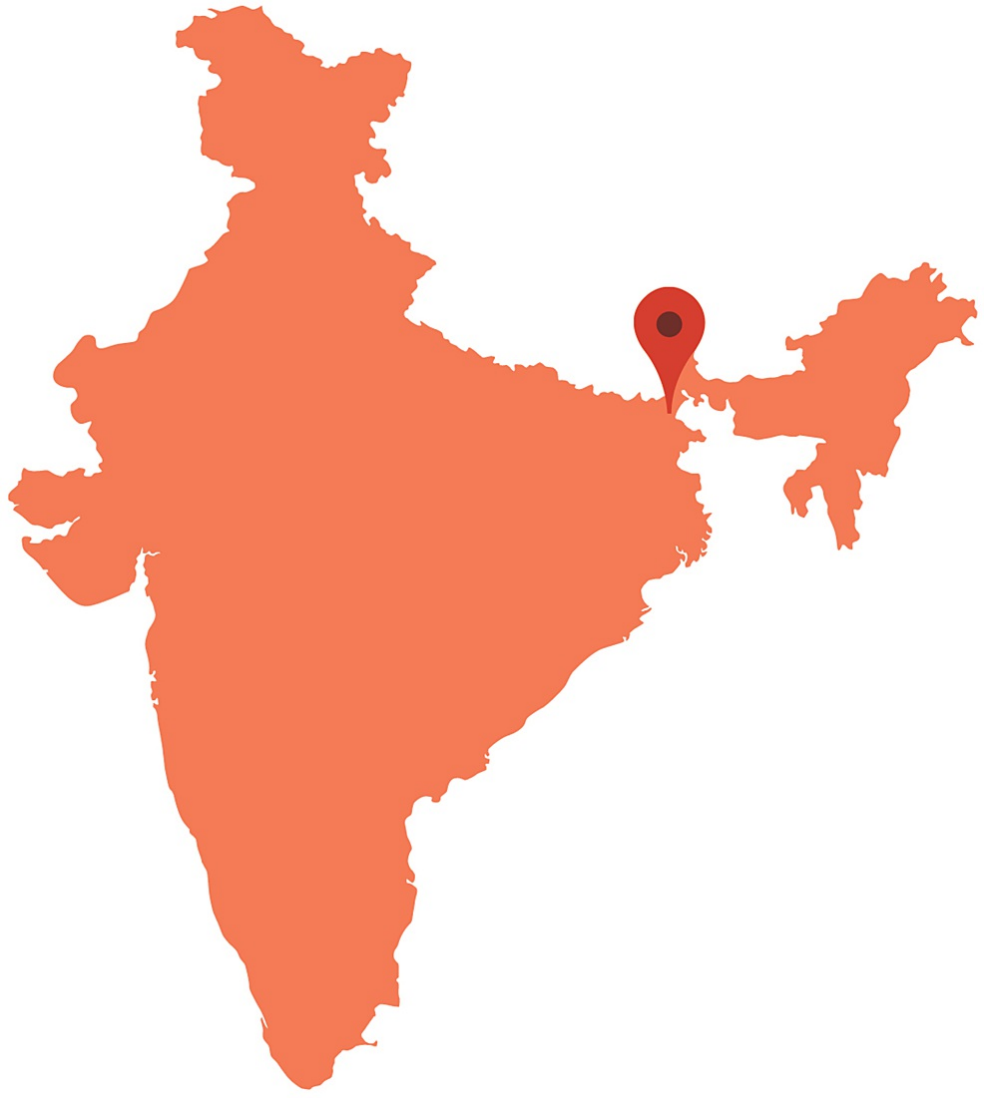
Position of the study site on the Indian map

This cross-sectional observational study was conducted in a tertiary care hospital situated in Bihar state of India. The area is a semi-urban area that caters mainly to people of rural and semi-urban areas. The location of the institution where the study was conducted is shown in a map in Figure 1. After obtaining permission from the Institutional Ethics Committee of Mata Gujri Memorial Medical College, Kishanganj, Bihar, India (reference number: IEC/06/2020), this study was conducted in the Department of Microbiology from January 2021 to December 2021.

Participants

All patients referred in this period to the Department of Microbiology from the Department of Dermatology and clinically provisionally diagnosed with superficial mycosis and sent for mycology were included in the study. For adult patients (age ≥ 18 years), written informed consent was obtained, and for patients <18 years of age, written informed consent was obtained from the parent of the patient, and verbal assent was obtained from the participant. Patients with any other comorbidities and having any other acute diseases or on any therapy (e.g., antifungal or topical steroids) for the diseases were excluded from the study.

Sample collection

Clinical history and demographics were captured in a pretested proforma. Samples were collected from nail clippings, skin scales, crusts, and easily pluckable hair. The lesions were scraped from the center to the edge of the infected area. The scraping was carried out such that it covered the whole diseased region. Using sterile tweezers, the base root section of the hair was removed. Scrapings from close to the nail bed, debris from beneath the nails, and nail clippings were all recovered in tinea unguium infections. Samples were gathered, folded, and brought to the laboratory in a thick black paper envelope.

Tests

The collected specimens underwent direct microscopic inspection after being wet and prepared with potassium hydroxide (KOH) (10% KOH for skin and hair and 40% KOH for nails). A test tube containing Sabouraud’s dextrose agar (SDA) with 0.05% chloramphenicol and 0.5% cycloheximide was then filled with specimens for inoculation. For up to four weeks, the infected specimens were incubated at 25°C. After four weeks, if there is no growth, then the material was deemed to be negative for fungal growth. For those where the growth was observed, the colony shape, pigmentation, fungal growth rate, and microscopy were used to identify the different fungal isolates.

Statistical analysis

Data were expressed in descriptive statistics as numbers and percentages. Categorical data were compared statistically using the chi-square test. For the entire test, we used GraphPad Prism 6.01 (GraphPad Software, San Diego, CA, USA). A p-value of <0.05 was considered statistically significant for this study.

## Results

Among the 330 patients, 186 (56.4%) were males and 144 (43.6%) were females. The majority of patients (24%) were from the age group of 21-30 years (Table 1). The youngest patient was of two years, and the highest age was 65 years. The majority of the cases (73%) were new, and 27% were relapsed. Among the 330 patients, diagnosis of dermatophytosis was confirmed by direct microscopy in 198 (66%) patients, and out of these patients, culture was positive in 68 (20.6%). Out of the 198 microscopically confirmed cases, we found 92 (46.4%) cases of tinea capitis (55 endothrix and 37 ectothrix), followed by 68 (34.3%) cases of tinea corporis. Age-wise distribution of the 198 microscopically confirmed cases of dermatophytosis with their clinical presentation is shown in Table 1.

**Table 1:** Age-wise distribution of microscopically confirmed dermatophytosis cases

Provisional diagnosis	Number (%)	Age of patients (years)						p-value
		1-10	11-20	21-30	31-40	41-50	>51	
		Number (%)						
Tinea corporis	68 (34.34)	3 (6)	7 (21.88)	42 (53.16)	11 (57.89)	2 (22.22)	3 (33.33)	<0.0001
Tinea capitis	92 (54.76)	43 (86)	12 (37.5)	30 (37.97)	2 (10.53)	4 (44.44)	1 (11.11)	<0.0001
Tinea unguium	11 (5.56)	0	1 (3.13)	2 (2.53)	3 (15.79)	1 (11.11)	4 (44.44)	0.31
Tinea faciei	5 (2.53)	3 (6)	1 (3.13)	0	0	1 (11.11)	0	0.46
Tinea pedis	6 (3.03)	0	3 (9.38)	2 (2.53)	1 (5.26)	0	0	0.61
Mixed	16 (8.08)	1 (2)	8 (25)	3 (3.8)	2 (10.53)	1 (11.11)	1 (11.11)	0.02
Total	198	50	32	79	19	9	9	<0.0001

Out of the 198 microscopically positive samples, 68 (34.34) samples showed growth of dermatophytes. *Trichophyton* was the predominant fungus isolated, and *Trichophyton mentagrophytes* was the most common species (Table 2).

**Table 2:** Culture results of the microscopically confirmed cases of dermatophytosis

Organism isolated	Number of patients (n = 198)	χ^2^, p-value
Trichophyton mentagrophytes	36 (52.9%)	187.8, <0.0001
Trichophyton rubrum	16 (23.5%)	
Trichophyton verrucosum	10 (14.7%)	
Epidermophyton	6 (8.8%)	
Contaminant fungi	101 (51.01%)	
No growth	29 (14.6%)	

Out of 330 patients, 223 (67.58%) patients were from the low socioeconomic group, and 194 (58.79%) had poor personal hygiene practices. Patient demographics are shown in Table 3.

**Table 3:** Patient demographics and socioeconomic factors The p-value is of the binomial test.

Socio-environmental factors	Yes (%)	No (%)	p-value*
Low socioeconomic group	223 (67.58)	107 (32.42)	<0.0001
Poor personal hygiene	194 (58.79)	136 (41.21)	0.002
Overcrowding	180 (54.55)	150 (45.45)	0.11
Fieldwork (farmer, construction worker)	162 (49.09)	168 (50.91)	0.78
Immigrant labors	158 (47.88)	172 (52.12)	0.47
Contact with animals	101 (30.61)	229 (69.39)	<0.0001
Multiple sexual contacts	98 (29.7)	232 (70.3)	<0.001

## Discussion

A class of fungus called dermatophytes mostly affects superficial tissues such as the skin, hair, and nails and can result in cutaneous mycoses [11]. Infections with dermatophytes are more common in underdeveloped countries. Dermatophyte infections have been observed in many parts of India and are thought to thrive in the hot, humid climate of tropical and subtropical regions. The current study emphasizes the clinicomycological profile of dermatophytoses due to the dearth of literature in the eastern part of India.

Our study included 330 cases of all age groups, and we found that the majority of the patients belonged to the age group of 21-30 years. Sharma et al. [7], Sarma et al. [12], and Kalita et al. [13] also reported the highest number of cases in the same age group. People in this group of age are frequently the most active and engaged in outdoor activities, which may be the primary cause of the increased frequency in this age group.

We found a higher prevalence in males, and this report is supported by other studies from India [7,13,14]. As compared to other studies done earlier, where cases of tinea corporis were high, in our study, we found tinea capitis to be the highest. The reason for this may be the prolonged covering of heads by field and construction workers for occupational reasons in this particular region. In India, males are more exposed to outdoor activities, leading to excessive sweating; this may be the underlying cause for the disparity in the occurrence of infection between sexes. The reason for lower incidence in females may be due to decreased reporting of patients to the clinics due to the attached social stigma in semi-urban areas. Only one recent study by Das et al. showed a higher incidence in females, which contradicts our finding [8].

*Trichophyton rubrum* had been identified in earlier Indian investigations as the most frequent cause of dermatophytosis [7,15]. However, a change in the pattern was seen in India over the past five years due to an increase in the incidence of *Trichophyton mentagrophytes* [16,17]. About two-thirds of illnesses were caused by *Trichophyton mentagrophytes*, according to a 2014 study from Himachal Pradesh [3]. Our study also reports *Trichophyton mentagrophytes* as the commonest species (52.9%) causing the infection.

Table 4 shows a comparison of various studies done at different times with their significant findings related to dermatophytosis.

**Table 4:** Comparative table showing significant observations by different studies related to dermatophytosis M: male; F: female

Study	Major affected age group (years)	Sex predilection	Commonest clinical presentation	Commonest organism
Sarma et al. (2007) [12]	21-30	Males (M:F = 3:1)	Tinea corporis (42%)	*Trichophyton rubrum* (47.54%)
Naglot et al. (2015) [14]	21-30	Males (M:F = 2.24:1)	Tinea corporis (34.82%)	*Trichophyton rubrum* (50.15%)
Kalita et al. (2019) [13]	21-30	Males (M:F = 2.1:1)	Tinea corporis (75%)	*Trichophyton mentagrophytes* (55%)
Das et al. (2020) [8]	18-40	Males (M:F = 1:1.78)	Tinea corporis (86.4%)	*Trichophyton verrucosum* (53.1%)
Singh et al. (2020) [17]	21-30	Males (M:F = 1.22:1)	Tinea corporis (65%)	*Trichophyton mentagrophytes* (79.2%)
Current study (2021)	21-30	Males (M:F = 1.3:1)	Tinea capitis (46.4%)	*Trichophyton mentagrophytes* (52.9%)

The prevalence of dermatophytosis in the population is also greatly influenced by socioeconomic factors, lifestyle choices, and migration. The current study examined the epidemiology of dermatophyte diseases in a semi-urban district of Eastern India. The majority of the patients were from the low socioeconomic group. Maintenance of poor personal hygiene is another potential factor responsible for dermatophytosis. Since this part of Bihar shares its border with West Bengal and has proximity to the state of Assam and Nepal, the immigration of laborers to and from the region also contributes to the aggravated spread of infection. Further studies are required to gain a better understanding of the epidemiology and the causative fungal species responsible for dermatophytoses in the entire state of Bihar and the eastern part of India.

Limitation

This is a hospital-based study conducted with a convenience sample. Hence, the generalization of the study result is limited. We could not perform antifungal susceptibility testing because of a lack of resources in the institution.

## Conclusions

Tinea capitis was the most common clinical presentation of dermatophytosis in a tertiary care hospital in Bihar, India. *Trichophyton mentagrophytes* was the most common organism found. Low socioeconomic status and poor personal hygiene were the factors associated with the high prevalence of dermatophyte infections in this region of India. Further detailed studies are required to understand the epidemiological factors and various fungal species responsible for dermatophytosis so that preventive and therapeutic measures can be taken to decrease the incidence.

## References

[1] Nweze EI, Eke IE (2018). Dermatophytes and dermatophytosis in the eastern and southern parts of Africa. Med Mycol.

[2] Kaufman G, Berdicevsky I, Woodfolk JA, Horwitz BA (2005). Markers for host-induced gene expression in Trichophyton dermatophytosis. Infect Immun.

[3] Bhatia VK, Sharma PC (2014). Epidemiological studies on dermatophytosis in human patients in Himachal Pradesh, India. Springerplus.

[4] Kumar GS, Kar SS, Jain A (2011). Health and environmental sanitation in India: Issues for prioritizing control strategies. Indian J Occup Environ Med.

[5] Noronha TM, Tophakhane RS, Nadiger S (2016). Clinico-microbiological study of dermatophytosis in a tertiary-care hospital in North Karnataka. Indian Dermatol Online J.

[6] Singh A, Masih A, Khurana A (2018). High terbinafine resistance in Trichophyton interdigitale isolates in Delhi, India harbouring mutations in the squalene epoxidase gene. Mycoses.

[7] Sharma R, Adhikari L, Sharma RL (2017). Recurrent dermatophytosis: a rising problem in Sikkim, a Himalayan state of India. Indian J Pathol Microbiol.

[8] Das S, De A, Saha R (2020). The current Indian epidemic of dermatophytosis: a study on causative agents and sensitivity patterns. Indian J Dermatol.

[9] Jain S, Kabi S, Swain B (2020). Current trends of dermatophytosis in eastern Odisha. J Lab Physicians.

[10] Saha I, Podder I, Chowdhury SN, Bhattacharya S (2021). Clinico-mycological profile of treatment-naïve, chronic, recurrent and steroid-modified dermatophytosis at a tertiary care centre in eastern India: an institution-based cross-sectional study. Indian Dermatol Online J.

[11] White TC, Findley K, Dawson TL Jr (2014). Fungi on the skin: dermatophytes and Malassezia. Cold Spring Harb Perspect Med.

[12] Sarma S, Borthakur AK (2007). A clinico-epidemiological study of dermatophytoses in Northeast India. Indian J Dermatol Venereol Leprol.

[13] Kalita JM, Sharma A, Bhardwaj A, Nag VL (2019). Dermatophytoses and spectrum of dermatophytes in patients attending a teaching hospital in Western Rajasthan, India. J Family Med Prim Care.

[14] Naglot A, Shrimali DD, Nath BK, Gogoi H, Veer V, Chander J, Tewari R (2015). Recent trends of dermatophytosis in Northeast India (Assam) and interpretation with published studies. Int J Curr Microbiol App Sci.

[15] Sharma M, Sharma R (2012). Profile of dermatophytic and other fungal infections in jaipur. Indian J Microbiol.

[16] Verma SB, Panda S, Nenoff P (2021). The unprecedented epidemic-like scenario of dermatophytosis in India: I. Epidemiology, risk factors and clinical features. Indian J Dermatol Venereol Leprol.

[17] Singh BS, Tripathy T, Kar BR, Ray A (2020). Clinicomycological study of dermatophytosis in a tertiary care hospital in eastern India: a cross-sectional study. Indian Dermatol Online J.

